# Mechanical Behavior of Recycled Aggregate Concrete-Filled Steel Tubular Columns before and after Fire

**DOI:** 10.3390/ma10030274

**Published:** 2017-03-09

**Authors:** Wenchao Liu, Wanlin Cao, Jianwei Zhang, Ruwei Wang, Lele Ren

**Affiliations:** College of Architecture and Civil Engineering, Beijing University of Technology, Beijing 100124, China; liuwenchao@emails.bjut.edu.cn (W.L.); zhangjw@bjut.edu.cn (J.Z.); wangruwei2014@emails.bjut.edu.cn (R.W.); 1154775846@emails.bjut.edu.cn (L.R.)

**Keywords:** recycled aggregate concrete, recycled aggregate concrete-filled steel tube, axial compression tests, fire exposure, loss of bearing capacity, mechanical behavior

## Abstract

Recycled aggregate concrete (RAC) is an environmentally friendly building material. This paper investigates the mechanical behavior of recycled aggregate concrete filled steel tube (RACFST) columns exposed to fire. Two groups of 12 columns were designed and tested, under axial compression, before and after fire, to evaluate the degradation of bearing capacity due to fire exposure. Six specimens were subjected to axial compression tests at room temperature and the other six specimens were subjected to axial compression tests after a fire exposure. The main parameters of the specimens include the wall thickness of the steel tube (steel content) and the type of concrete materials. Several parameters as obtained from the experimental results were compared and analyzed, including the load-bearing capacity, deformation capacity, and failure characteristics of the specimens. Meanwhile, rate of loss of bearing capacity of specimens exposed to fire were calculated based on the standards EC4 and CECS28:90. The results show that concrete material has a large influence on the rate of loss of bearing capacity in the case of a relatively lower steel ratio. While steel content has little effect on the rate of loss of bearing capacity of concrete-filled steel tube (CFST) columns after fire, it has a relatively large influence on the loss rate of bearing capacity of the RACFST columns. The loss of bearing capacity of the specimens from the experiment is more serious than that from the calculation. As the calculated values are less conservative, particular attention should be given to the application of recycled aggregate concrete in actual structures.

## 1. Introduction

Recycled aggregate concrete (RAC) is a type of concrete material that is made from recycled concrete aggregate (RCA) obtained from waste concrete by cleaning, crushing, and grading [1,2]. The emergence of RAC not only greatly reduces the demand for natural aggregate, but also reduces the environmental pollution caused by construction waste [3]. At present, scholars have conducted a lot of research on recycled aggregate concrete. In 1997, the physical and mechanical properties of recycled aggregate concrete were studied by Topçu et al. [4], the results showed that as the amount of WCA (waste concrete aggregate) increases, density, workability, Schmidt hardness, ultrasound velocity, and compressive strength decrease. Sagoe-Crentsil et al. [5] studied the performance of recycled coarse aggregate concrete in 2001 and showed that the performance of commercially produced recycled concrete is similar to that of natural concrete. In 2005, the mechanical properties of recycled aggregate concrete with different coarse aggregate replacement ratio were studied by Xiao et al. [6] and the result showed that the peak strain of RAC is higher than that of normal concrete. When RCA replacement percentage equals 100%, the peak strain was increased by 20%. In 2007, Evangelista et al. [7] studied the material properties of recycled fine aggregate concrete and the results showed that a replacement rate of fine aggregate up to 30% is safe and reliable. Rahal [8] studied the mechanical properties of concrete with recycled coarse aggregate, the results showed that reduced modulus of elasticity and compressive strength are normally associated with the use of recycled concrete aggregate. In 2010, the durability of recycled fine aggregate concrete, including chloride ion permeability and carbonation reaction, was studied by Evangelista et al. [9], and the result showed that water absorption increases linearly with the replacement ratio. In 2015, Behnood et al. [10] studied the modulus elasticity of recycled aggregate concrete using M5 model tree algorithm and the results show that the model developed using the M5 algorithm has accuracy over 80 percent, which is well above the accuracy of the other models. In 2016, mechanical properties of recycled concrete subjected to high temperature were studied by Gales et al. [11]. Meanwhile, Li and Xiao et al. [12] studied the dynamic compressive behavior of recycled coarse aggregate concrete and the results showed that the critical strain of RAC changed insignificantly with an increase in strain rate. In 2016, Bendimerad et al. [13] studied the recycled concrete plastic shrinkage and cracking sensitivity coefficient and the results showed that replacement of fine aggregate by recycled fine aggregate has a large influence on the early age strength and cracking sensitivity of recycled aggregate concrete.

The concept of recycled aggregate concrete was first proposed in 1950s. With extensive research, recycled concrete is no longer a novel technology, and the relevant research is mainly focused on the application of recycled concrete [14]. Flexural performance of recycled concrete beams was studied by Sato et al. [15] and Kang et al. [16]. Their results showed that recycled concrete can be used to cast beams if coarse aggregate replacement rate is limited to 30%; the flexural performance of recycled concrete beams and ordinary concrete beams are basically the same. Cao et al. [17] studied the flexural performance of high strength recycled aggregate concrete slabs with steel bar truss. The results showed that the cracking load and the ultimate load of high strength recycled aggregate concrete slabs were comparable to those of ordinary high-strength concrete slab. The seismic behavior of recycled concrete shear wall was systematically studied by Cao et al. [18,19,20] and Zhang et al. [21,22], which provide experimental and theoretical basis for the application of recycled concrete in shear walls.

In the application of recycled concrete column structure, Konno et al. [23,24] reported the results of an experimental study and provided a theoretical analysis of recycled concrete column encased by steel tube in 1997. Meanwhile, the results show that the confining effect has great influence on the bearing capacity of the specimen. To this end, some scholars studied the different confining effects between concrete and steel or other materials [25,26,27,28,29,30,31,32,33,34]. Because of its excellent mechanical performance and environment friendliness, recycled aggregate concrete-filled steel tubular (RACFST) column has become an active research topic globally. Yang et al. [35] investigated the axial compression performance of RACFST columns, and showed that the failure mode of RACFST column is in the form of overall instability damage. Mohanraj et al. [36] studied the behavior of steel tubular stubs and slender columns filled with recycled aggregates concrete, and compared the experimental results with those calculated using Eurocode4 (EC4).

Fire is one of the most frequent and widespread disasters in a structure. Once fire occurs in a building, the bearing capacity of the building will be greatly reduced. Fire may cause a continuous collapse type accident, resulting in serious loss of life and property. A study [37] foresaw that RACFST columns will be gradually applied to the high-rise building in the future, so it is necessary to investigate the fire resistance of RACFST columns. At present, correlative research reports are few. Yang et al. [38] studied the axial load performance of recycled aggregate concrete-filled steel tubular short column after being subjected to high temperature, and proposed a simplified formula to calculate axial compressive bearing capacity. Chen et al. [39] studied the mechanical behavior of eccentrically loaded recycled aggregate concrete-filled circular steel tube columns after being subjected to high temperature. Luo et al. [40] studied the fire resistance of recycled concrete-filled steel tubular columns. These studies have focused on the mechanical properties of RACFST columns under constant temperature or on the fire resistance limit of RACFST columns under heat-stress coupling. However, study on the residual bearing capacity of RACFST columns after a fire exposure has not been reported.

To this end, two groups of 12 RACFST specimens are designed in this paper. The main parameters are steel ratio (wall thickness of steel tube) and the type of concrete materials. One group comprises control specimens that are subjected to axial compression test, and the other group is subjected to axial compression test after being exposed to ISO-834 standard heating. The mechanical properties of the RACFST columns are studied at room temperature and after fire.

## 2. Testing Program

### 2.1. Test Parameters

**Figure 1:**
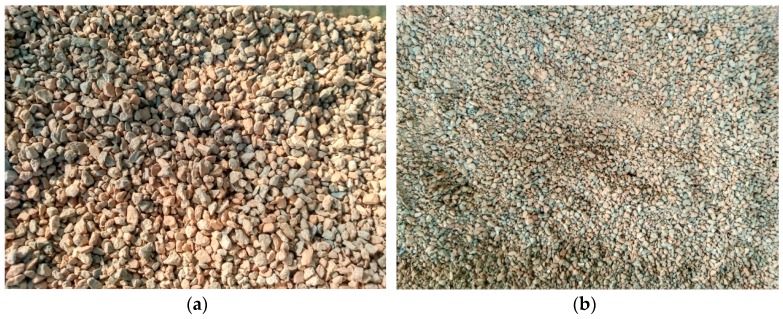
The aggregate of RAC: (**a**) the coarse aggregate size is 5–10 mm; and (**b**) the fine aggregate size is 0–5 m.

**Figure 2:**
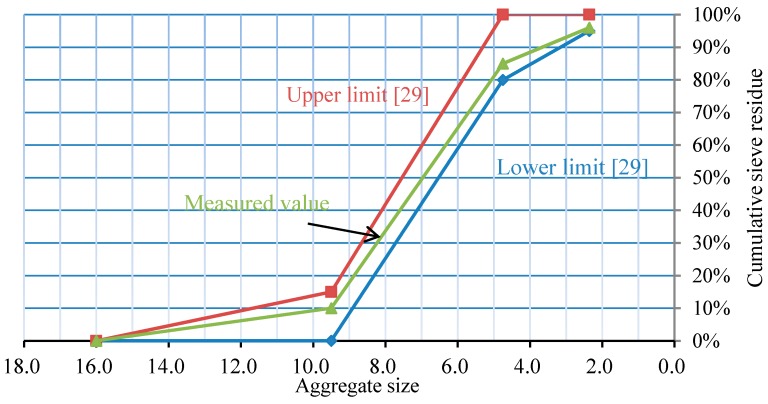
Particle size distribution curve of the coarse aggregate in RCA.

The length of specimens is 500 mm; the steel tube was seamless tube with wall thickness of either 4 mm or 5 mm; and the diameter of the specimen is 500 mm. The concrete material types were divided into three types: (1) ordinary fine aggregate concrete (FAC); (2) semi-recycled concrete with the replacement rate of recycled coarse aggregate as 100%; and (3) fully recycled concrete, that is, the coarse and fine aggregate are fully replaced by recycled coarse aggregate and fine aggregate. The experimental parameters of concrete for the first group are shown in Table 1, and for the second groups are shown in Table 2. The size of coarse aggregate for the above three types of concrete materials was 5–10 mm, and the size of fine aggregate was 0–5 mm. Recycled aggregates were processed by Beijing Shougang Co., Ltd. (Beijing, China). Figure 1 shows a photograph of the recycled coarse and fine aggregate. Necessary parameters of the recycled coarse aggregate are in line with the standard GB/T 25177-2010 [41]. Figure 2 shows the particle size distribution curve of the coarse aggregate in RCA. It is worth noting that the particle size distribution of normal aggregates was 5–25 mm. The mix proportion of ordinary concrete and recycled aggregate concrete was 1:0.49:2.28:2.28:0.21:0.21 (cement:water:sand:pebble/recycled-pebble:fly ash:mineral powder), and the design strength of concrete was C40 (40 MPa).

In Table 1 and Table 2, FC represents the column which experienced a fire; H represents a hollow steel tube (not filled with concrete); N represents a steel tube filled with ordinary concrete; and R represents a steel tube filled with recycled concrete. Moreover, Ra is the semi-recycled concrete with 100% recycled coarse aggregate; Rb is fully recycled concrete with 100% recycled aggregate; T is the wall thickness of the steel pipe, where T1 means the thickness of steel tube is 4 mm, and T2 means the thickness of steel tube is 5 mm; D is the outside diameter of the pipe; L is the length of the specimen; L/D is the aspect ratio; RP_RCA_ is the replacement percentage of coarse aggregate in the recycled concrete; and RP_RFA_ represents the replacement percentage of fine aggregate in recycled concrete.

### 2.2. Material Properties

The standard cube compressive strength test was performed on standard concrete cubes (100 mm side with adjustment coefficient of 0.95 [42]). There are three samples for each concrete cubes. The average compressive strength of concrete cubes was obtained as shown in Table 3. It is worth noting that the coefficient of variation (standard deviation/average Mean) was less than 10%.

Before the fire test, the concrete cubes (each cubes has three samples), which had been cured in advance, were put into a fire stove at 0–1000 °C for an hour. After the fire exposure, the cubes were tested for compressive strength. The average compressive strengths of concrete cubes after fire exposure are shown in Table 4. In addition, the coefficient of variation was less than 10%.

**Figure 3:**
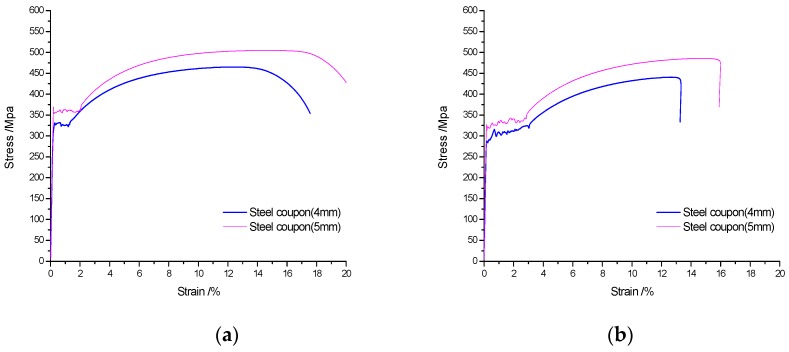
Stress–strain curves of the steel. (**a**) The curves before fire; (**b**) The curves after fire.

Coupons from the steel tube were tested for tensile strength before and after fire exposure according to the standard GB/T 228-2002 [43]. The dimensions of steel coupons are 4 (thickness) × 10 (width) × 80 (length) and 5 (thickness) × 10 (width) × 80 (length). Each steel coupon has three samples. Tensile strength results are shown in Table 5, in which *f*_y_ is the yield strength at room temperature; *f*_u_ is the ultimate strength at room temperature; and *δ* is the breaking strain of the steel coupon. Correspondingly, *f*_y_-fire is the yield strength after fire; *f*_u_-fire is the ultimate strength after fire; and *δ*-fire is the breaking strain of the steel coupon after fire. The stress–strain curves of steel coupons before and after fire are show in Figure 3.

### 2.3. Construction of Test Specimens

**Figure 4:**
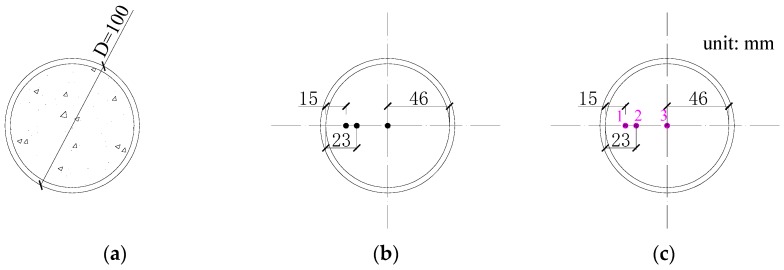
Arrangement of the thermocouples. (**a**) Specimen size; (**b**) Positions of thermocouples; (**c**) Numbers of thermocouples.

**Figure 5:**
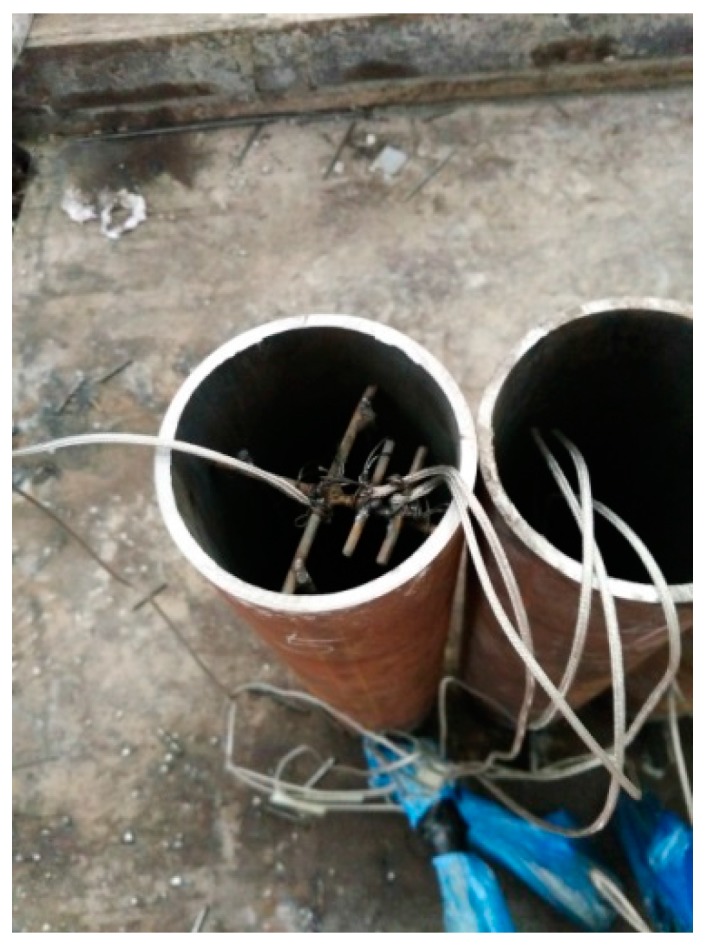
Steel positioning skeleton.

**Figure 6:**
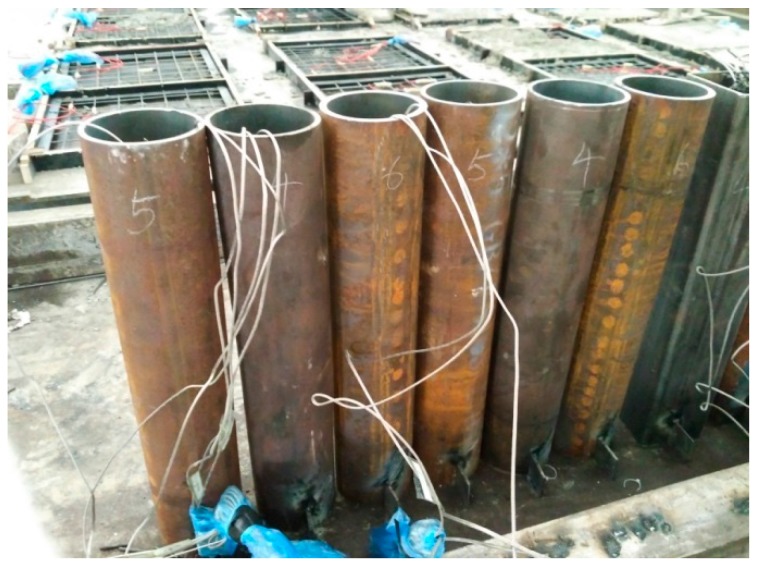
Construction of the specimens.

For the first group of specimens (C-H-T1, C-H-T2, C-N-T1, C-N-T2, C-Ra-T1 and C-Rb-T2), the specimens were subjected to axial compression test directly. For the second group of specimens (FC-N-T1, FC-N-T2, FC-Ra-T1, FC-Ra-T2, FC-Rb-T1 and FC-Rb-T2), due to the need for fire resistance test, three K-type thermocouples were arranged in a circular tube based on the principle of symmetry. The arrangement of the sectional drawing is shown in Figure 4. Three measuring points were arranged in depth 250 mm, that is, the middle of the specimen. In order to accurately position the thermocouples, a steel positioning skeleton was set, as shown in Figure 5. The specimens were constructed as shown in Figure 6.

## 3. Performance of RACFST under Axial Compression at Room Temperature

### 3.1. Test Set-Up and Loading Program

**Figure 7:**
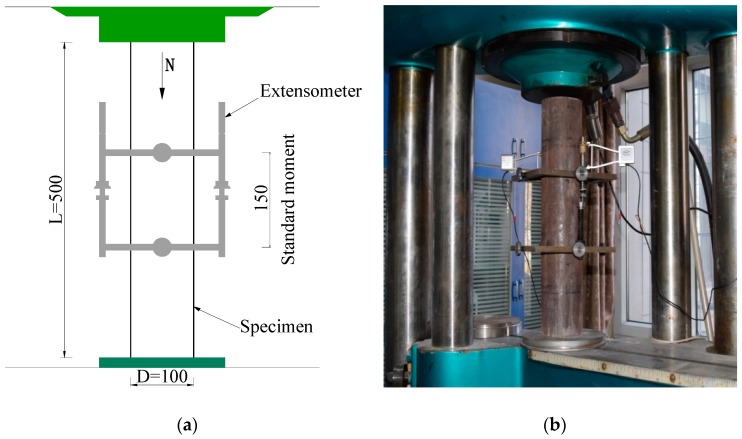
Test set-up. (**a**) Sketch map of the test; (**b**) Scene photos of the test.

The experiment was conducted in the mechanics laboratory of Beijing University of Technology using a 100-tonne capacity hydraulic testing machine, WE-100. To ensure that the measured deformation values could truly reflect the deformation of the column, an extensometer (with standard distance of 150 mm) was installed around the middle of the columns. The displacement of the specimen was measured by a displacement meter, and the test specimen was arranged as shown in Figure 7. Before beginning loading, a test specimen was physically aligned by preloading to ensure that the specimen was under axial compression, and the preload value did not exceed 1/10th of the estimated ultimate load. The displacement control method was used and the loading rate is 2 mm/min.

### 3.2. Failure Modes of Specimens at Room Temperature

**Figure 8:**
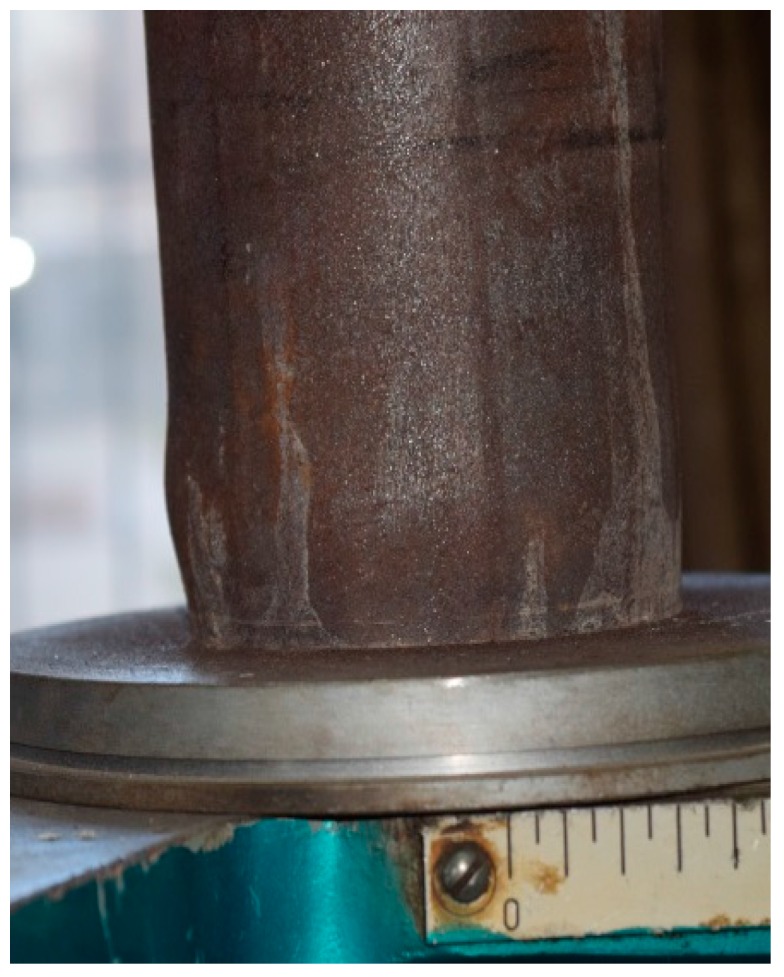
Deformation (expansion) of the specimens.

**Figure 9:**
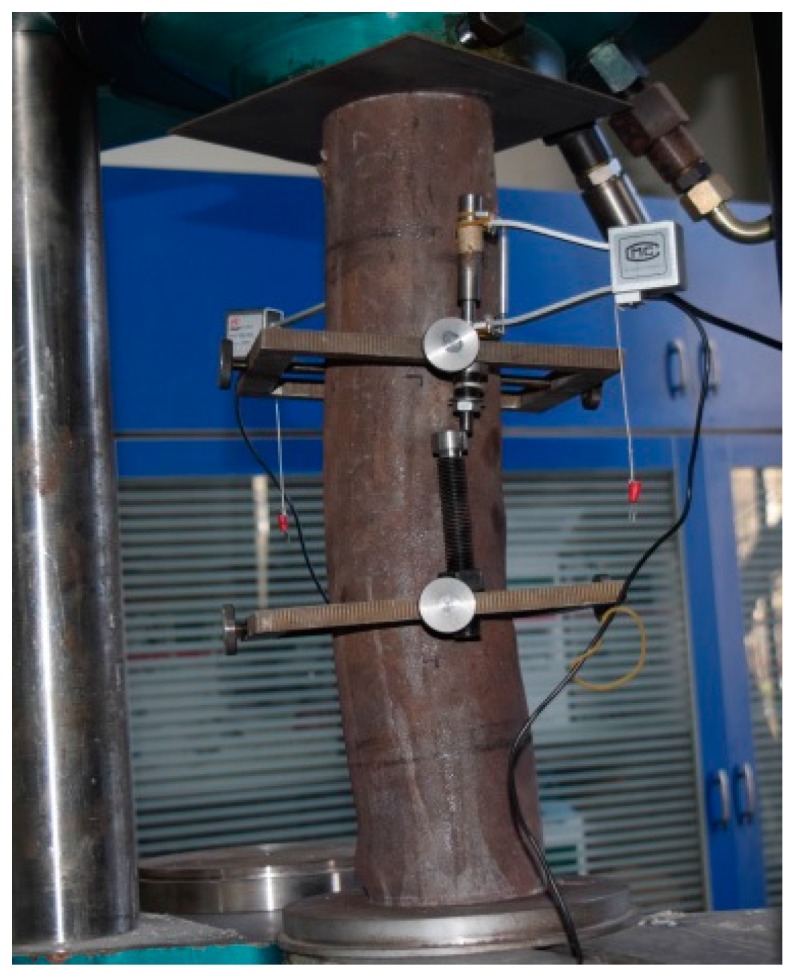
Failure modes of C-N-T1.

Specimen C-N-T1 is taken as a typical example to describe the failure process of the specimen. The deformation of the specimen is not significant during the initial loading; the main phenomenon that was observed for the steel surface was the peeling of rusted material. When the axial load reached 650 kN, the steel tube began to yield, and the rate of deformation of the steel tube was accelerated. When the load reached 780 kN, the specimen exhibited an elastic-plastic platform. When the load reached 890 kN, the lower part of the specimen showed relatively larger expansion as shown in Figure 8. At this point the central and upper expansion is not significant, but the central bending deformation is relatively serious. As the load continued to increase, the extent of deformed area increased. When the load reached 940 kN, the bottom of the specimen appeared as a drum package, and the middle and upper part of the specimen exhibited a large bending deformation, as shown in Figure 9. After that, the load began to slow down and the middle part of the specimen buckled on one side. The bending deformation and compression deformation of specimen were quite serious at that moment. Taking into account the safety factor, the test was stopped and the specimen was thought to be destroyed.

**Figure 10:**
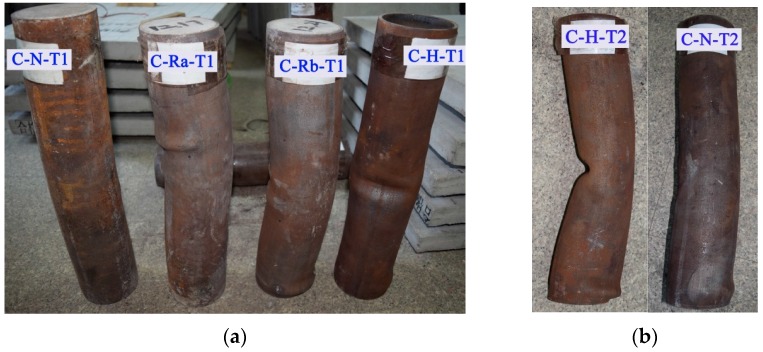
Failure modes of the specimens. (**a**) The wall thickness of steel tube is 4 mm; (**b**) The wall thickness of steel tube is 5 mm.

Figure 10 shows the failure modes of the six specimens from the first group; all six specimens experienced bending damage to one side of the column. It can be seen that the damage of the concrete-filled steel tubular (CFST) columns is relatively small, whereas that of the RACFST is relatively large, especially for the fully recycled aggregate concrete specimens. For specimens C-H-T1 and C-H-T2, which are not filled with concrete, when the load reached the ultimate load of the specimen, the middle of the steel pipe rapidly yielded, forming an obvious depression.

Table 6 shows yield load *F*_y_, peak load *F*_u_, and their corresponding axial deformations are as follows: the axial deformation of yield load Δ_y_, the axial deformation peak load of Δ_u_. Each load has a measured value (MV) and relative value (RV).

With the same wall thickness of steel tube, the yield load of C-H-T1 specimen is relatively low. The difference between the yield loads of C-N-T1, C-Ra-T1 and C-Rb-T1 is only 2.6%–2.9%. The results show that concrete material has little effect on the yield loads of specimens. Under the yield load, the deformation of hollow steel tube specimen is the largest. The axial deformation of C-Rb-T1 specimen is 12.3% and 11.0% higher than that of C-N-T1 and C-Ra-T1, respectively. Under the peak load, the axial deformation of C-Rb-T1 is also the largest. The reason is that the elastic modulus of recycled concrete (Rb) is relatively low. It is shown that the concrete material has a great influence on the axial deformation of the specimen.

With the same concrete materials, the yield and peak load of C-N-T2 were 8.8% and 14.9% higher than that of C-N-T1. Meanwhile, the axial deformation under peak load of C-N-T2 is obviously smaller than that of C-N-T1. It is shown that with the increase of the wall thickness of the steel tube, the axial stiffness of the specimen under the peak load is obviously improved, and the increase of axial stiffness of specimen under yield load is not significant.

### 3.3. Load–Deformation Relationship of RACFST at Room Temperature

**Figure 11:**
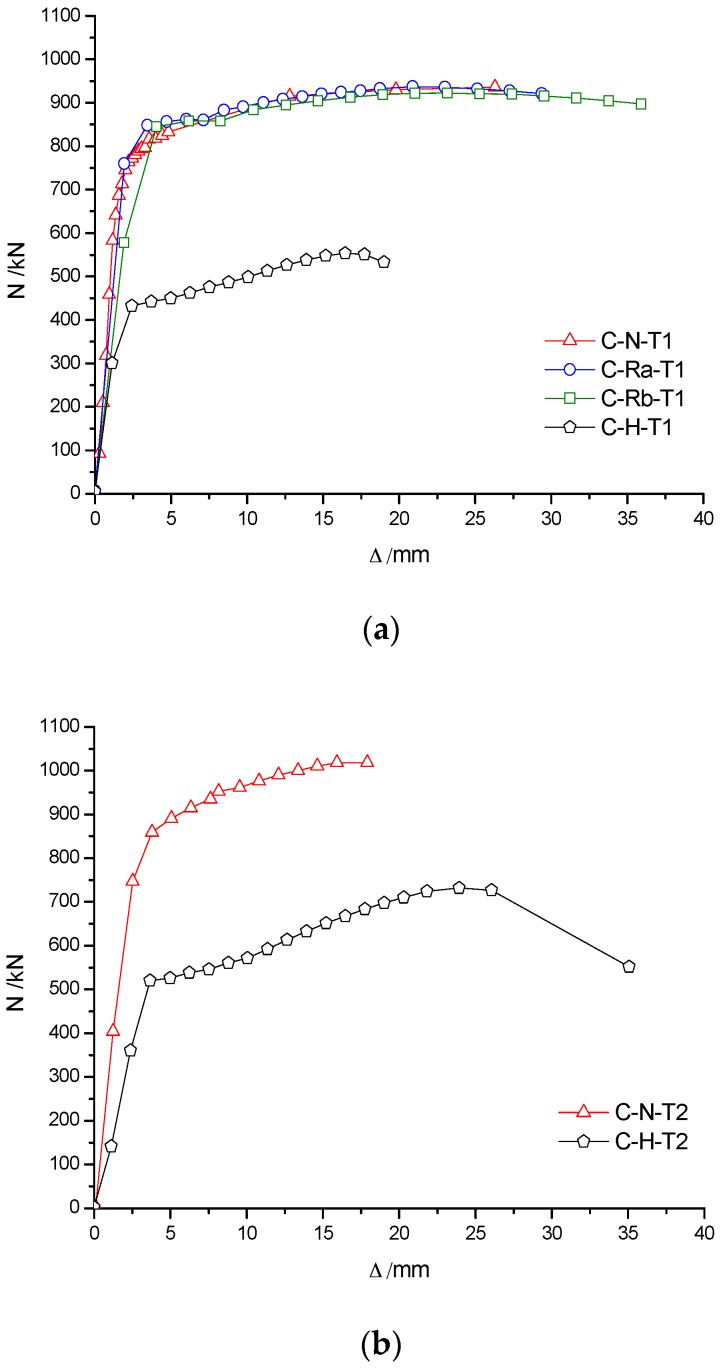
Influence of concrete material on N-Δ curves. (**a**) The wall thickness of the steel tube is 4 mm; (**b**) The wall thickness of the steel tube is 5 mm.

**Figure 12:**
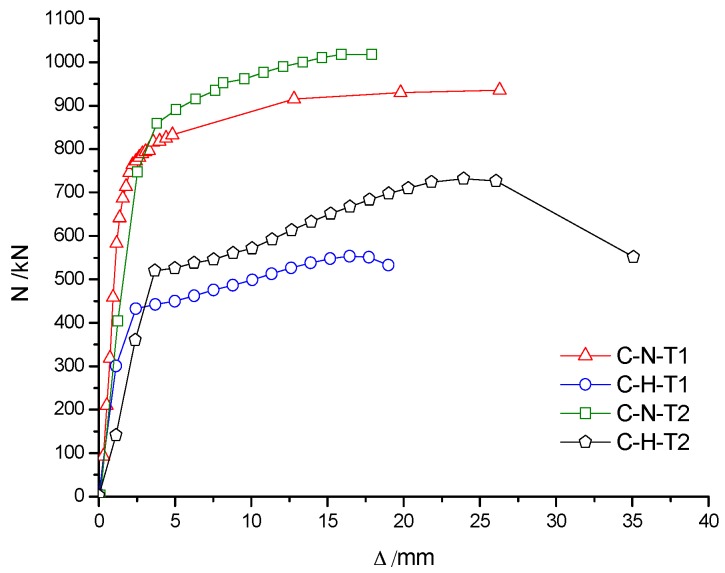
Influence of wall-thickness on N-Δ curves.

Figure 11a shows that the load–deformation curves of C-N-T1, C-Ra-T1 and C-Rb-T1 specimens were almost coincident with each other in the case of the same wall thickness of the steel pipe. The peak loads for C-N-T1, C-Ra-T1 and C-Rb-T specimens were, respectively, 937 kN, 935 kN and 922 kN. The maximum difference in the peak loads was only 15 kN, which indicated that the concrete material type had little influence on the bearing capacity of RACFST columns. However, the peak load of specimen C-H-T1 (unfilled concrete) was 548 kN, which is about 42% less than the former three values, indicating that concrete infill can greatly improve the bearing capacity and ductility of RACFST columns. The main reason is attributed to the fact that both steel tube and concrete can fully contribute in sharing the axial compressive load. Figure 11b shows that the peak load of C-N-T2 specimen was 1018 kN and the peak load of C-H-T2 was 732 kN (28% less than that of C-N-T2). It is shown that with an increase in wall thickness of the steel tube, the contribution of infill concrete on the bearing capacity of the specimen was reduced. Therefore, it is suggested that infill concrete is extremely useful for thin-walled steel tubes in improving the bearing capacity of the column. Figure 12 shows that, for the same concrete material, the peak load of C-N-T2 was approximately 81 kN higher than that of C-N-T1, and of C-H-T2 was approximately 184 kN higher than that of C-H-T1 (respectively, increased by 8% and 25%). Therefore, the wall thickness of the steel tube has a large influence on the bearing capacity of the specimens, especially for the hollow steel tube specimens.

### 3.4. Load–Axial Strain Relationship of RACFST at Room Temperature

**Figure 13:**
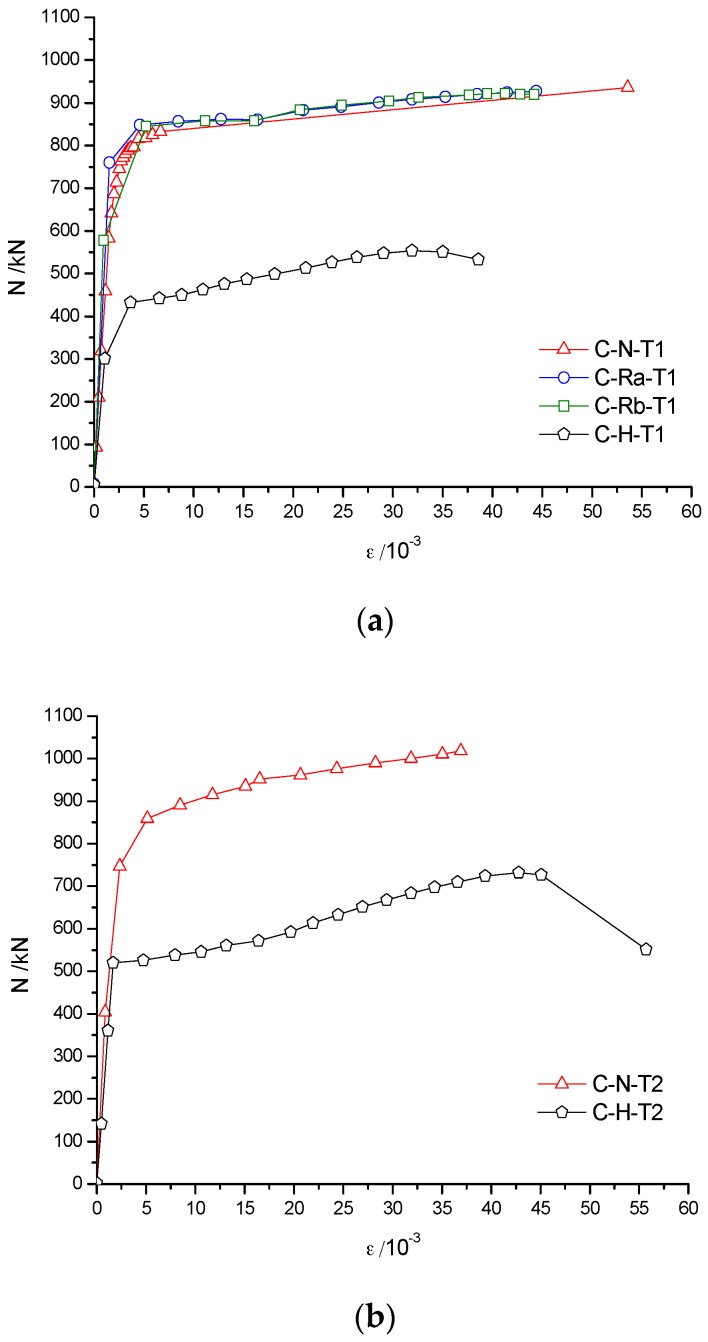
Influence of concrete materials on N-*ε* curves. (**a**) The wall thickness of steel tube is 4 mm; (**b**) The wall thickness of steel tube is 5 mm.

Because the deformation of one point is random, and the deformation of different points is not uniform. In paper, the development of the mean strain of the central area (150 mm of the 500 mm) was monitored. Figure 13a,b shows that the axial load vs. average longitudinal strain (N-*ε*) curve of the specimens can be divided into two stages, namely, the elastic stage and the plastic hardening stage. When the wall thickness of the steel pipe was 4 mm, the curves of the elasticity curves of C-N-T1, C-Ra-T1 and C-Rb-T1 were almost coincident, and the strain did not change with the load, which is due to the higher axial stiffness. On the other hand, because of its low axial stiffness, the strain of C-H-T1 was slightly larger than that of the above-mentioned three specimens. A similar observation was made for the specimens with the wall thickness of 5 mm. When the curve entered into the plastic hardening stage, strain increased rapidly with an increase in load. Concrete type has little effect on the axial rigidity of the specimen.

**Figure 14:**
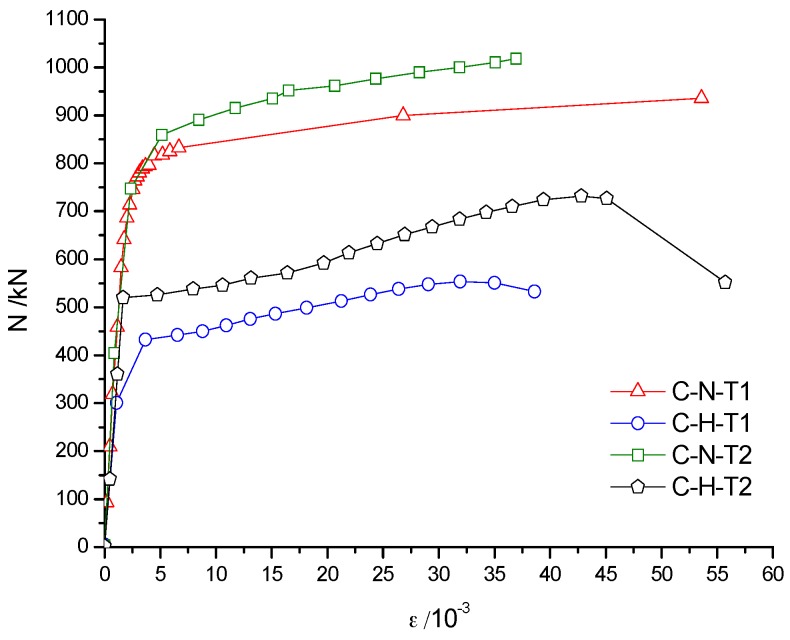
Influence of wall-thickness on N-*ε* curves.

When the specimens had the same concrete materials (Figure 14), the N-*ε* curves of C-N-T1 and C-N-T2 were basically the same in the elastic phase, but the curves started to deviate in the early stage of plasticity. As shown in Figure 14, the specimen with smaller wall thickness had larger strain and lower bearing capacity, which is attributed mainly to the difference in axial rigidity.

## 4. The Fire Test and the Post-Fire Axial Loading Test of RACFST

### 4.1. General Situation of the Fire Test

Six specimens from the second group were subjected to fire before performing the axial loading test. The main parameters were the concrete material type and the wall thickness of the steel pipe (steel ratio). The distribution of temperature field and the failure modes of RACFST columns were investigated after fire.

**Figure 15:**
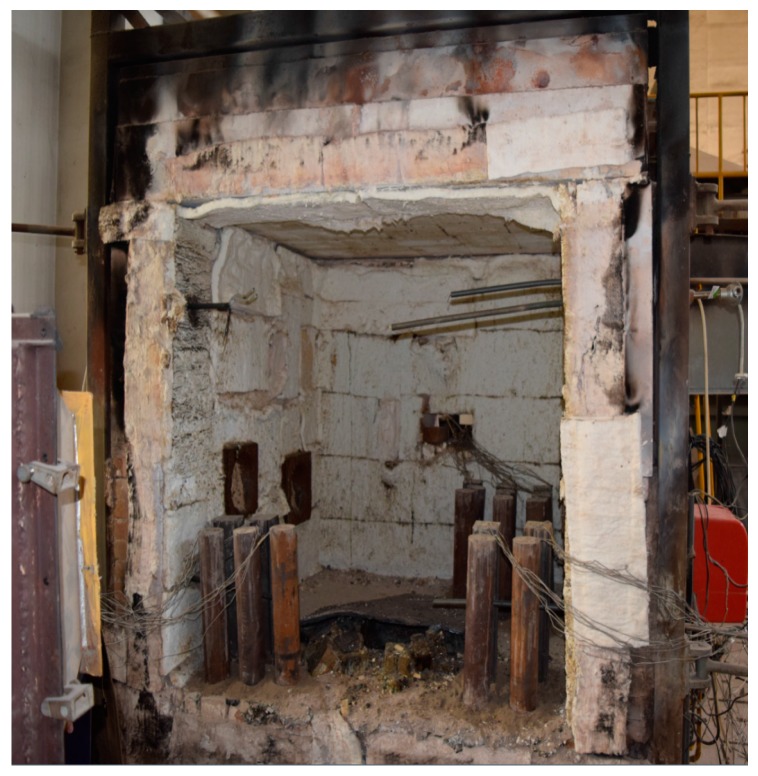
Vertical fire furnace.

**Figure 16:**
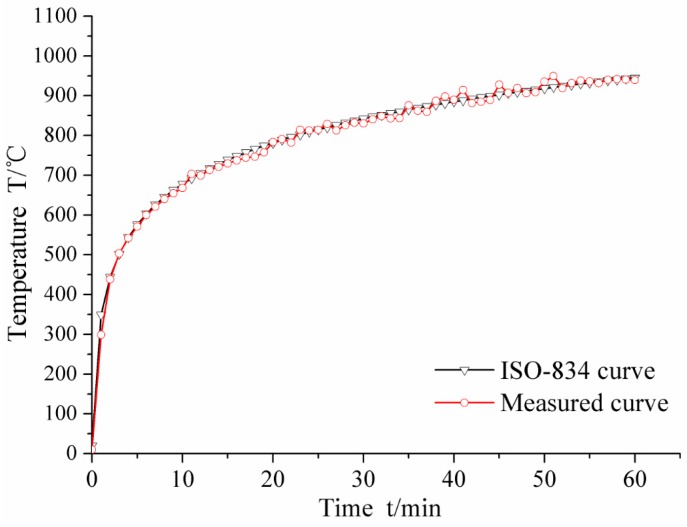
The furnace temperature curve.

The fire test was carried out in the fire laboratory of China Academy of Building Research (CABR). Six specimens were put into a small vertical fire furnace as shown in Figure 15. The rock wool was used to protect the top surface and bottom surface of the columns free from fire. ISO-834 standard temperature curve (from 0 to 1000 °C) was used, and the burning time was set as 1 h. The average measured temperature curve was as shown in Figure 16. Natural cooling was used for cooling the specimens to room temperature.

**Figure 17:**
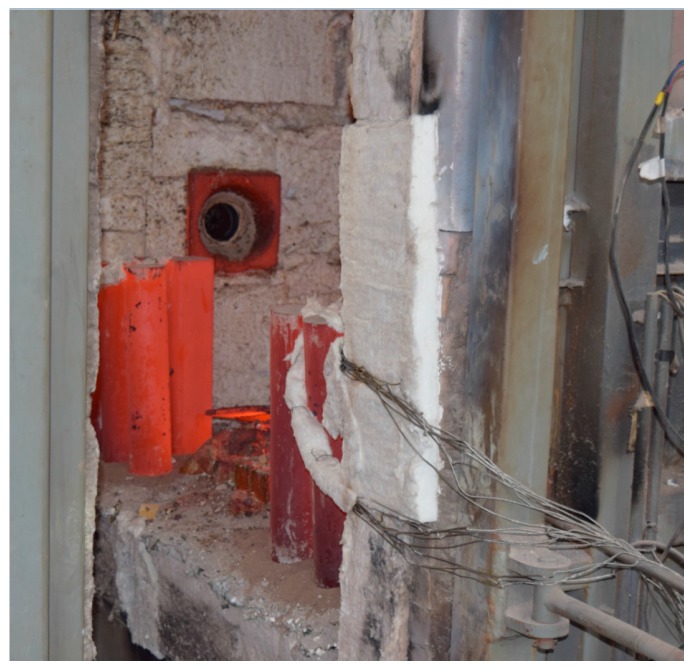
The end of the fire test.

**Figure 18:**
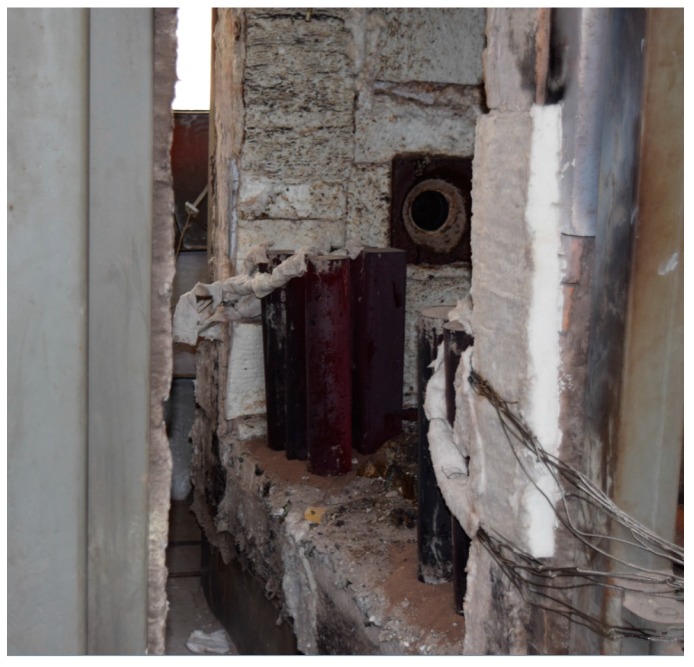
Natural cooling.

Figure 16 shows that the average measured temperature curve coincided with the ISO-834 curve. This indicates that the test condition was ideal, and hence, provided a reliable initial condition for the post fire axial compression test. In the combustion process, white water vapor could be seen outside the fire furnace during the first 10–15 min, indicating that the water in the concrete of the specimen was evaporated. Some cracking sound could be heard from the specimen, which was caused by the decarburization. When burning time reached to 1 h, the fire furnace was opened. As shown in Figure 17, the burnt specimen was very red because of the high temperature. With a decrease in temperature, the color turned into black, as shown in Figure 18. As the aggregate size is small (coarse aggregate size was 5–10 mm), no spalling was found during the fire test. Thus, the integrity of the specimens is good after fire.

### 4.2. Monitoring of Internal Temperature of Specimen

**Figure 19:**
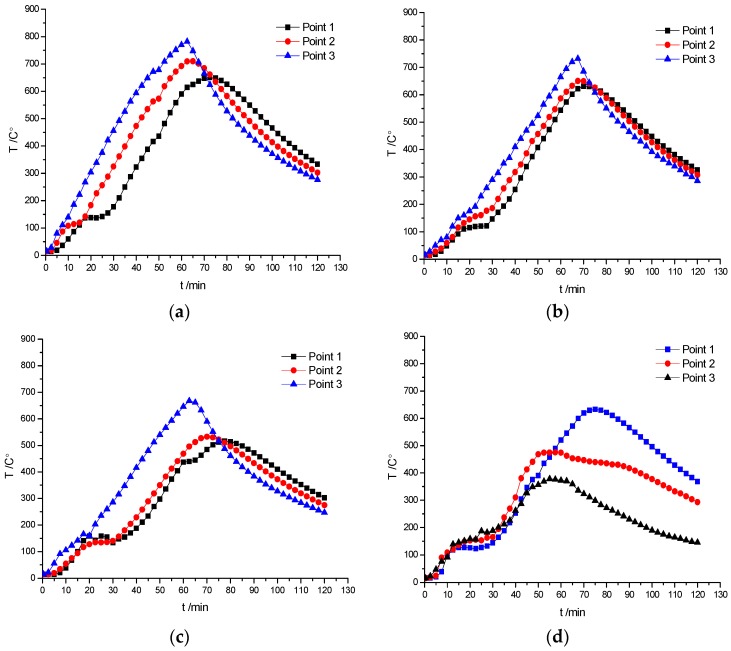
Temperature–time curve of specimens. (**a**) FC-N-T1; (**b**) FC-Ra-T1; (**c**) FC-Rb-T1; (**d**) FC-Rb-T2.

In order to analyze the internal temperature field of the specimens, the temperature–time curves were recorded by the thermocouples as shown in Figure 19.

Figure 19 shows that once the temperature reached 100–150 °C (corresponding to the time of 25–30 min), it stayed nearly constant for a while, which indicates that the water in the specimen was volatilized and the heat was taken away. Temperature in FC-N-T1 and FC-Rb-T1 specimens reached the maximum value in 62.5 min, which indicated that the temperature-rise process had some delay. It means that even though the fire was stopped after 60 min, the internal temperature of the specimen still increased for few minutes before entering into the descending section. It can be seen that the peak temperature was first reached at the lateral measuring point 1 (Figure 4) followed by the measuring points 2 (Figure 4) and 3 (Figure 4). This observation indicates that the closer the measuring point from the specimen center, the longer was the time to attain the peak temperature. The maximum temperature of FC-N-T1 was 782.5 °C and of FC-Rb-T1 was 667.3 °C. The difference of 115.2 °C indicates that the thermal conductivity of recycled aggregate concrete was relatively lower. The steady temperature platform of FC-Rb-T1 was 30 min, which was 5 min longer than that of FC-N-T1. It can be seen that the fire-resistance property of recycled concrete is superior to that of ordinary concrete.

### 4.3. Failure Modes of Specimen after Fire

**Figure 20:**
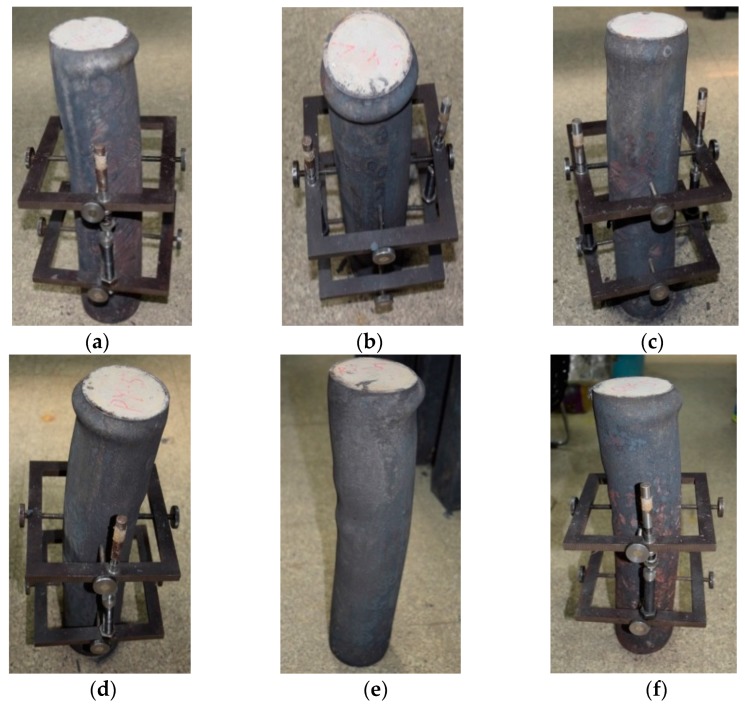
Failure modes of specimens: bending failure. (**a**) C-N-T1; (**b**) C-Ra-T1; (**c**) C-Rb-T1; (**d**) C-N-T2; (**e**) C-Ra-T2; (**f**) C-Rb-T2.

In the early stage of loading, both the displacement and the strain increased slowly with the load, the rust on the surface of the specimen began to fall off, the upper part of the steel column showed bulging but the deformation of the lower part of the column was not obvious. When the loading reached to 55%–65% of the ultimate load, the displacement and strain increased rapidly, the lateral expansion of the specimen was visible, the column began to warp to one side, and the top of the steel tube deformed seriously. When the load increased to the ultimate load, the extent of drum-shaped deformation was increasing, and the middle part of the specimen was severely buckled. After the ultimate load was exceeded, the longitudinal displacement and strain continued to increase, but the bearing capacity of the steel column decreased slightly. The specimen failure occurred finally in the form of buckling. The failure modes of the specimens are shown in Figure 20.

### 4.4. Load–Deformation Relationship of RACFST after Fire

**Figure 21:**
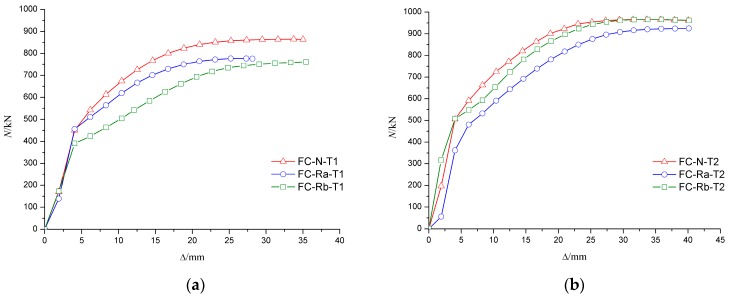
Influence of concrete material on N-Δ curve. (**a**) The wall thickness of steel tube is 4 mm; (**b**) The wall thickness of steel tube is 5 mm.

**Figure 22:**
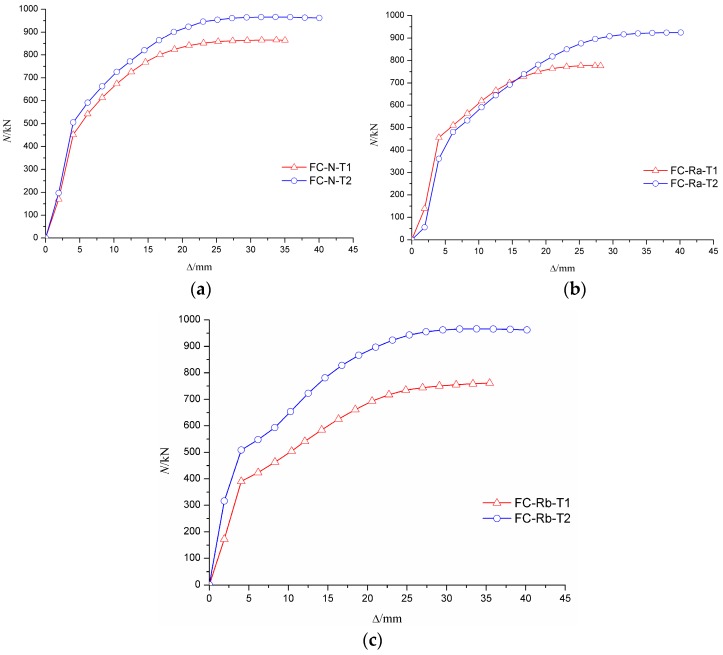
Influence of wall-thickness on N-Δ curves. (**a**) Fine aggregate concrete; (**b**) Recycled aggregate concrete (RP_RCA_ = 100%); (**c**) Recycled aggregate concrete (RP_RCA_ = 100% and RP_RFA_ = 100%).

It can be seen from the curve of axial load–axial displacement (N-Δ) in Figure 21 and Figure 22 that the axial rigidity of the specimen was almost constant in the early stage of loading. When the column began to buckle on one side, the curve was markedly changed and the axial rigidity decreased rapidly. At this time, the bearing capacity of the specimen began to decrease, but the descent process was relatively slow. The results showed that the ductility of the RACFST was still good even after the fire because of the constraint provided by the steel tube.

Figure 21a shows that, for the specimens with wall thickness of 4 mm, the ultimate load of C-N-T1 was approximately 10% and 12% higher than that of C-Ra-T1 and C-Rb-T1, respectively. The results show that the deterioration on the bearing capacity of RACFST was more serious than that of CFST. For the specimens with wall thickness of 5 mm, the difference in ultimate bearing capacity of the RACFST and CFST specimens was, however, reduced to 4%, as shown in Figure 21b. These observations indicated that concrete material was no longer the main factor when the wall thickness was increased. As shown in Figure 22, the specimens with the wall thickness of 5 mm were 11%, 16% and 21% stronger than the specimens having same concrete material but having 4 mm thick wall. This indicates that wall thickness has a large influence on the ultimate bearing capacity of the specimen.

### 4.5. Load–Axial Strain Relationship of RACFST after Fire

**Figure 23:**
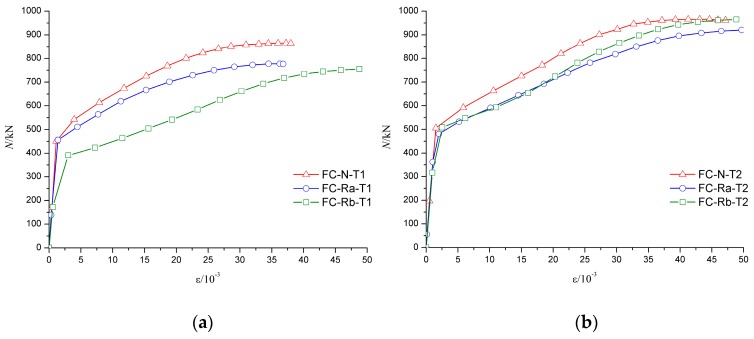
Influence of concrete material on N-*ε* curves. (**a**) The wall thickness of steel tube is 4 mm; (**b**) The wall thickness of steel tube is 5 mm.

**Figure 24:**
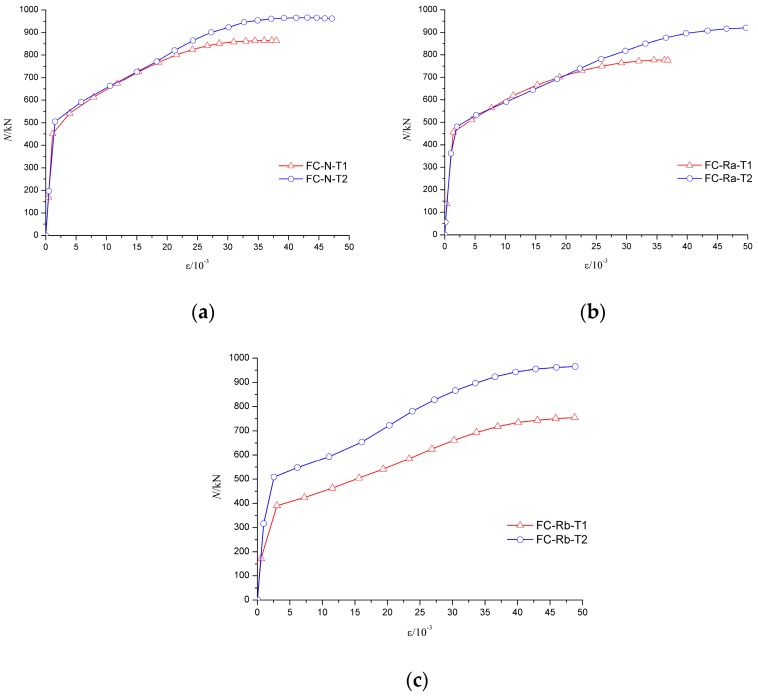
Influence of wall-thickness on N-*ε* curves. (**a**) Fine aggregate concrete; (**b**) Recycled aggregate concrete (RP_RCA_ = 100%); (**c**) Recycled aggregate concrete (RP_RCA_ = 100% and RP_RFA_ = 100%).

The axial force-average longitudinal strain (N-*ε* curve) of specimen can be used to analyze the deformation of the specimens, especially the deformation of the middle part of the specimens. As shown in Figure 23 and Figure 24, due to a relatively large axial stiffness, the average longitudinal strain of the specimens developed slowly in the early stage of failure. As the middle part of the specimen buckled, the longitudinal strain increased rapidly until the specimen failure.

Figure 23a shows that the trend of the axial force–average longitudinal strain (N-*ε*) curve was basically coincident in the elastic stage for FC-N-T1 and FC-Ra-T1 specimens. Because of the low axial stiffness, the elastic phase of FC-Rb-T1 was relatively short, which means that even with a relatively smaller load, the middle of the specimen began to yield. Figure 23b shows that the trend of the elastic stage of the specimens with the wall thickness of 5 mm was basically identical, which shows that the influence of concrete type on the elastic stage of the specimen became smaller with an increase in wall thickness of the steel tube. Figure 24 shows that wall thickness has a great impact on the ultimate load of the specimen.

## 5. Comparative Analysis of Bearing Capacity of RACFST Column before and after Fire

### 5.1. Analysis of the Load–Deformation Curve

**Figure 25:**
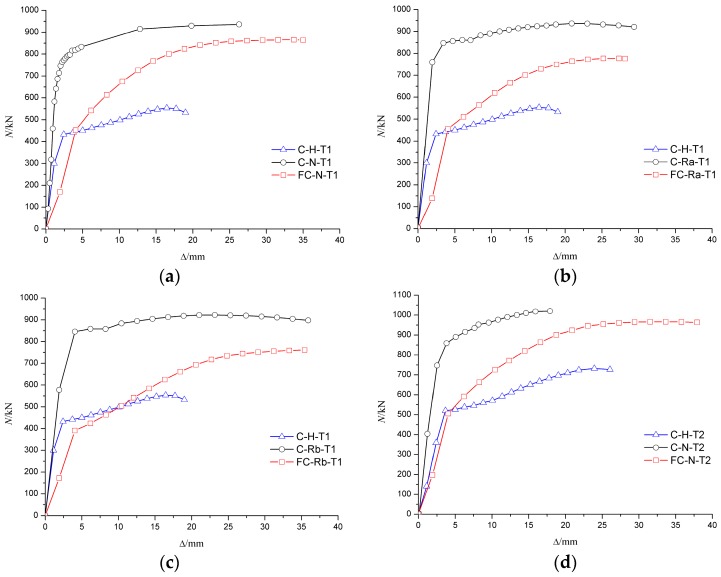
Load–deformation curves of specimens before and after fire. (**a**) Fine aggregate concrete (wall thickness is 4 mm); (**b**) Recycled aggregate concrete-Ra (wall thickness is 4 mm); (**c**) Recycled aggregate concrete-Rb (wall thickness is 4 mm); (**d**) Fine aggregate concrete (wall thickness is 5 mm).

Figure 25 shows that the axial deformation of the specimen after fire increased significantly. The slope of the red line (specimen that was subjected to fire) in the load–deformation curve was smaller than that of the blue line (specimen without concrete infill) and the black line (specimen that was not subjected to fire), thus indicating that the axial compression stiffness of the specimen was weakened after the fire. Compared to the specimens that were not subjected to fire, the bearing capacity of specimens was decreased after fire. For instance, the bearing capacity of FC-N-T1 decreased by 7.6%, of FC-Ra-T1 decreased by 17.1% and of FC-Rb-T1 decreased by 17.5%. The results show that the bearing capacity of recycled concrete specimen was decreased seriously after fire, but the ductility was still acceptable. Therefore, it is feasible to reinforce and repair the recycled concrete column after fire.

Compared to the specimen C-N-T2 that was not subjected to fire, the bearing capacity of FC-N-T2 decreased by only 5.3%, which indicated that an increase in wall thickness of the steel tube could reduce the damage of the infill concrete. Therefore, a reasonable increase in the wall thickness of a steel tube can not only increase the axial compressive bearing capacity of specimens under normal temperature, but also improve the residual bearing capacity of specimens after fire.

### 5.2. Analysis of the Load–Strain Curve

**Figure 26:**
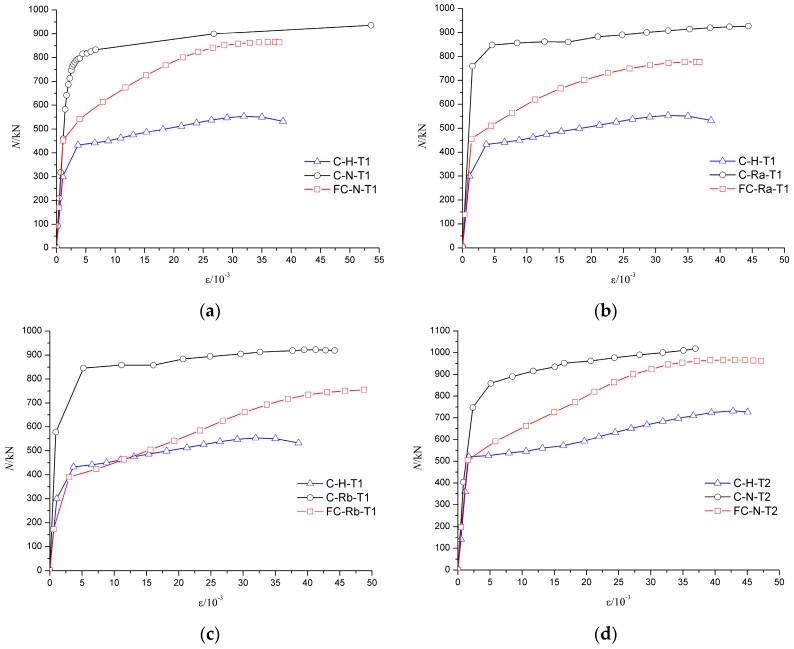
Load–strain curves of specimens before and after fire. (**a**) Fine aggregate concrete (wall thickness is 4 mm); (**b**) Recycled aggregate concrete-Ra (wall thickness is 4 mm); (**c**) Recycled aggregate concrete-Rb (wall thickness is 4 mm); (**d**) Fine aggregate concrete (wall thickness is 5 mm).

The trend of load–strain curves of C-N-T1 (FC-N-T1) and C-Ra-T1 (FC-Ra-T1) were basically identical for the same wall thickness before and after the fire. The results showed that the semi-recycled concrete with 100% coarse aggregate had good mechanical properties before and after fire, but the ultimate bearing capacity of C-Ra-T1 was degraded after fire, as shown in Figure 26a,b. As can be seen in Figure 26c, the axial compressive stiffness of the fully recycled concrete specimen, with 100% replacement by coarse and fine aggregates, was severely damaged by fire, and its elastic phase was also relatively short. It is suggested that in the case of a small load, the middle part of the steel tube will be greatly deformed, and even experience bulging. Therefore, this kind of recycled concrete material is not recommended for actual projects. Figure 26a,d shows that when the steel tube yielded (*ε* = 5 × 10^−3^), the load of FC-N-T2 and FC-N-T1 was not much different, but the bearing capacity of FC-N-T2 could continue to rise for a long time. The results show that the wall thickness has a great influence on the bearing capacity of the specimen both before and after the fire.

### 5.3. Analysis of Bearing Capacity before and after Fire

In order to analyze the degradation of bearing capacity of specimens, the bearing capacities before and after fire were compared. Regardless of the impact of concrete materials (concrete material has little effect on the ultimate bearing capacity of the specimen at room temperature, see Section 4), the bearing capacity of the specimen at room temperature was calculated. The bearing capacity as per EC4 [44] is expressed by Equation (1).

(1) $$N_{u}=\eta_{2}A_{a}f_{y}/\gamma_{M0}+\left[1+\eta_{1}(t/D)(f_{y}/f_{c})\right]A_{c}f_{c}/\gamma_{c}$$

where $\eta_{1}$ is 4.575; $\eta_{2}$ is 0.775; $A_{a}$ is the sectional area of steel tube; $f_{y}$ is the standard value of steel strength; $f_{c}$ is the standard value of concrete strength; $A_{c}$ is the sectional area of concrete; e is the load eccentricity, taken as 0.00; D is the external diameter of specimen; and t is the wall thickness of the steel tube.

For $\bar{\lambda }=0.047\leq 0.5,\;e=0\leq D/10$ (the wall thickness is 4 mm), the constraint effect of steel tube on the core concrete was considered by introducing the parameters $\eta_{1}$ and $\eta_{2}$. Moreover, $\bar{\lambda }$ is the relative slenderness ratio and can be calculated by Equation (2), where $N_{uk}$ is the standard value of plastic axial bearing capacity; and $N_{cr}$ is the elastic critical value.

(2) $$\bar{\lambda }=\sqrt{N_{uk}/N_{cr}}$$

χ is the reduction factor for the relative slenderness ratio, and can be taken as 1.00 for $\bar{\lambda }=0.047\leq 0.2$.

The bearing capacity as per CECS 28:90 [45] is expressed by Equations (3) and (4) where $N_{0}$ is the bearing capacity of the concrete-filled steel tubular short column; $\varphi_{1}$ is the reduction factor of slenderness ratio and can be calculated by Equation (5); $\varphi_{b}$ is the reduction factor of load eccentricity, and can be taken as 1.00; and θ is the confinement index and can be calculated by Equation (6).

(3) $$N_{u}=\varphi_{1}\varphi_{b}N_{0}$$

(4) $$N_{0}=f_{c}A_{c}(1+\sqrt{\theta }+\theta )$$

In Equations (5) and (6), $l_{e}$ the equivalent calculation length of the column and can be taken as the length of specimen *L*.

(5) $$\varphi_{1}=\left\{ \begin{array}{ll} 1-0.115\sqrt{l_{e}/D-4} & l_{e}/D>4\\ 1 & l_{e}/D\leq 4 \end{array}\right.$$

(6) $$\theta =f_{a}A_{a}/f_{c}A_{c}$$

The results are shown in Table 7 where *N_u_*_,*e*_ represents the test result of the ultimate bearing capacity of the specimens under normal temperature; *N^f^_u_*_,*e*_ represents the test result of the ultimate bearing capacity of the specimens after fire; and *N_u_*_,*c*_ represents the calculated ultimate bearing capacity of the specimens under normal temperature.

When calculating the bearing capacity of the specimens, the standard values were used for the necessary parameters. It can be seen that the bearing capacity of the specimens which experienced fire was about 82.6%–92.3% of that before fire, indicating that the bearing capacity of the specimens decreased significantly after fire. Especially for specimens produced by recycled concrete, the bearing capacity was markedly decreased. Therefore, RACFST column should be properly protected from fire in actual structures, such as by adding external protective layer covering the concrete surface.

The calculated results based on EC4 and CECS28: 90 standards are close to the experimental values, with a discrepancy of 2.99%–3.60%. This indicates that the standard equations can be used to calculate the ultimate bearing capacity of RACFST columns under axial compression when at normal temperature. The calculated values are lower than the experimental values, so the calculated results are conservative.

**Figure 27:**
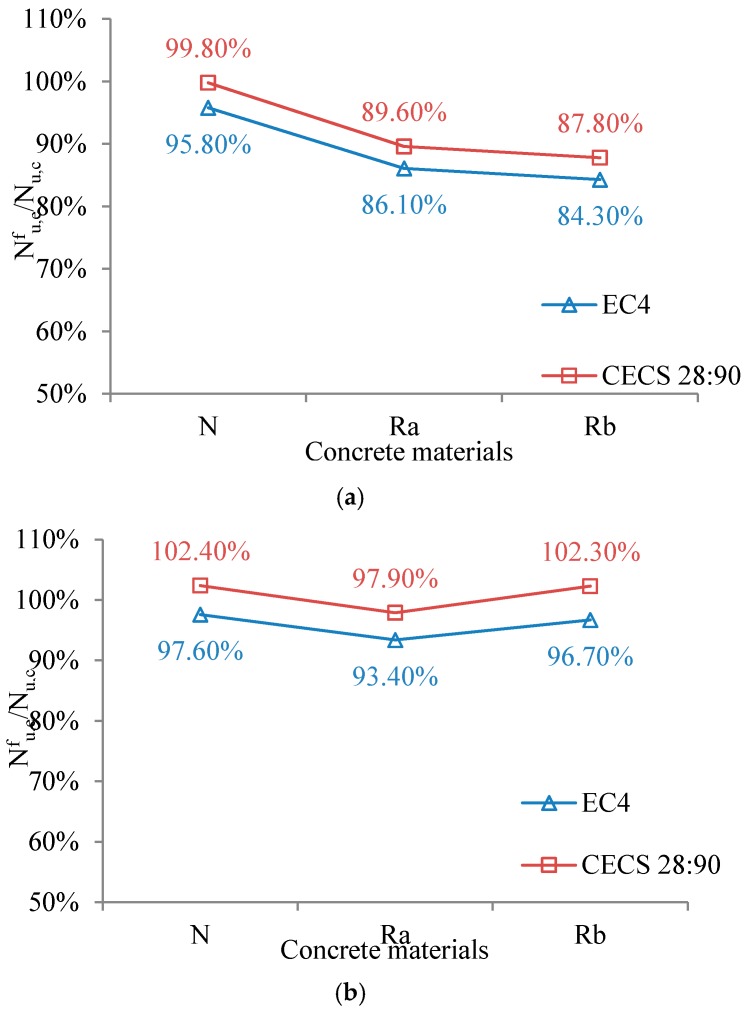
Influence of concrete materials. (**a**) The wall thickness of steel tube is 4 mm; (**b**) The wall thickness of steel tube is 5 mm.

**Figure 28:**
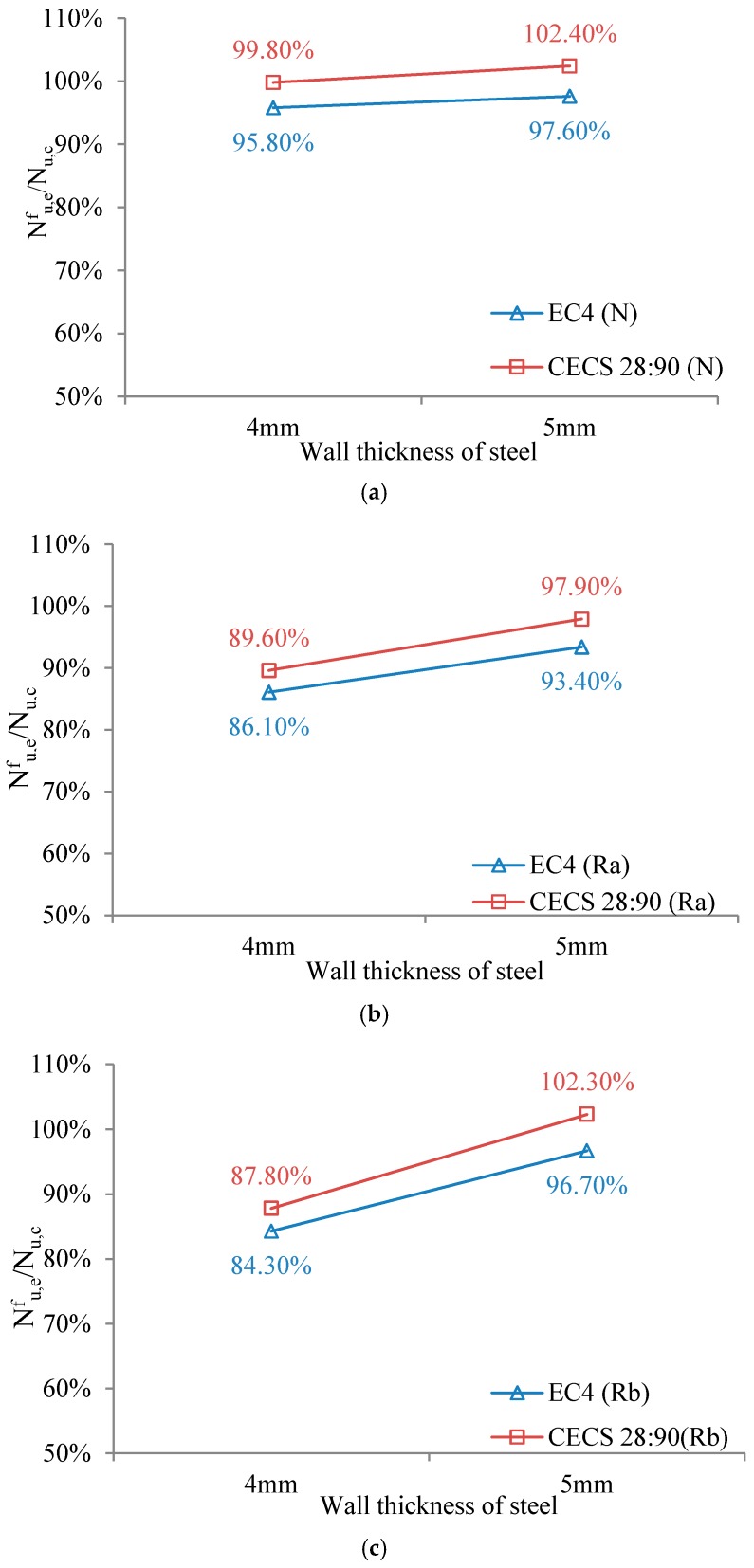
Influence of wall thickness of steel tube. (**a**) Fine aggregate concrete; (**b**) Recycled aggregate concrete (RP_RCA_ = 100%); (**c**) Recycled aggregate concrete (RP_RCA_ = 100% and RP_RFA_ = 100%).

In order to further analyze the degradation of bearing capacity of specimens after fire, relationships between the wall thickness, concrete material and the ratio of bearing capacity $N_{u,e}^{f}$/$N_{u,c}$ of specimens were proposed as shown in Figure 27 and Figure 28. Figure 27a shows that when the wall thickness of the steel pipe was 4 mm, the ratio of the bearing capacity $N_{u,e}^{f}$/$N_{u,c}$ of the specimen tended to decrease, which indicates that concrete material has a large influence on the rate of loss of bearing capacity of RACFST columns. As shown in Figure 27b, when the wall thickness of the steel tube was 5 mm, the ratio of the bearing capacity of the specimen was not significantly reduced, which indicates that concrete material has little effect on the rate of loss of bearing capacity after fire when wall thickness is relatively large.

Figure 28a shows that the wall thickness of steel tube has little effect on the rate of loss of bearing capacity of CFST columns after fire. Figure 28b,c shows that the wall thickness of steel tube has a relatively large influence on the rate of loss of bearing capacity of RACFST columns after fire. As the standard calculated values are conservative, the loss of bearing capacity of the actual structure after fire can indeed be more serious than indicated by calculation results.

## 6. Conclusions

In this paper, two groups of 12 recycled aggregate concrete-filled steel tubular (RACFST) columns were experimentally studied. Six specimens were subjected to axial compression tests under normal temperature, and the other six specimens were subjected to axial compression tests after fire. The failure modes and the degradation of bearing capacity of specimens were analyzed. The specific conclusions are as follows:

(1) The specimen failure occurred finally in the form of buckling. The failure mode of a recycled concrete column specimen which experienced fire was similar to that without fire. The N-Δ curve changed linearly in the early stage and then the axial stiffness of the specimen decreased rapidly after yielding. It is worth noting that the bearing capacity and deformation ability of specimens were weakened after fire; the RACFST columns were damaged seriously, but the overall ductility of specimen was still good.
(2) A temperature platform (the temperature is maintained at 100–150 °C) was observed in the temperature field of the interior of recycled concrete-filled steel tube columns and of ordinary concrete-filled steel tube columns. For concrete-filled steel tube column, the maximum temperature of FC-Rb-T1 was 14.7% lower than that of FC-N-T1, which indicates that the tendency of rise in internal temperature of RACFST column was low.
(3) For the specimens which experienced fire, when the wall thickness of the steel pipe was relatively small, concrete material had a large influence on the bearing capacity. However, with an increase in wall thickness of the steel tube, the influence of concrete material was diminished. For identical concrete material, the ultimate bearing capacity of the steel tube with wall thickness of 5 mm was 11% higher than that of 4 mm thick wall (ordinary concrete N) [16% (when coarse aggregate was 100% replaced by recycled aggregate, Ra) and 21% (when coarse and fine aggregate were 100% replaced by recycled aggregate, Rb)]. The wall thickness of steel pipe was the main factor to determine the bearing capacity of RACFST column.
(4) The axial compressive stiffness of specimens with 100% replacement of coarse and fine aggregates was severely damaged. Meanwhile, its elastic phase was relatively short, indicating that in the case of relatively smaller bearing capacity, the middle region of the steel tube showed large deformation, or even exhibited a drum package. Therefore, considering the safety of structures, it is not recommended to use recycled concrete material with 100% replacement of coarse and fine aggregate.

## Figures and Tables

**Table 1:** Experimental parameters of first group.

Specimen	D/mm	t/mm	L/mm	L/D	RP_RCA_/%	RP_RFA_/%
C-H-T1	100	4	500	5	-	-
C-H-T2	100	5	500	5	-	-
C-N-T1	100	4	500	5	0	0
C-N-T2	100	5	500	5	0	0
C-Ra-T1	100	4	500	5	100	0
C-Rb-T1	100	4	500	5	100	100

**Table 2:** Parameters of second group.

Specimen	D/mm	t/mm	L/mm	L/D	RP_RCA_/%	RP_RFA_/%
FC-N-T1	100	4	500	5	0	0
FC-N-T2	100	5	500	5	0	0
FC-Ra-T1	100	4	500	5	100	0
FC-Ra-T2	100	5	500	5	100	0
FC-Rb-T1	100	4	500	5	100	100
FC-Rb-T2	100	5	500	5	100	100

**Table 3:** Experimental results on the mechanical properties of concrete.

Type of Concrete	Concrete Design Strength (MPa)	Cube Compressive Strength (MPa)	Elastic Modulus (MPa)
N	C40/40.00	40.10	32,958
Ra	RC40/40.00	41.15	31,553
Rb	RC40/40.00	38.81	29,865

**Table 4:** Mechanical properties of concrete after fire.

Type of Concrete	Cube Compressive Strength (MPa)
N-fire	17.51
Ra-fire	16.32
Rb-fire	15.68

**Table 5:** Test results of steel plate properties.

Steel Coupons (mm)	*f*_y_ (MPa)	*f*_u_ (MPa)	*δ* (%)	*f*_y_-Fire (MPa)	*f*_u_-Fire (MPa)	*δ*-Fire (%)
4 × 10 × 80	326	469	16.6	287	444	13.3
5 × 10 × 80	347	513	19.3	325	498	15.9

**Table 6:** Experimental results of yield load, and peak load and their corresponding axial deformation.

Specimen	F_y_: kN		F_u_: kN		Δ_y_		Δ_u_	
	MV	RV	MV	RV	MV	RV	MV	RV
C-H-T1	455.53	1.000	553.48	1.000	5.53	1.000	16.44	1.000
C-N-T1	818.93	1.798	935.89	1.691	3.37	0.609	23.30	1.417
C-Ra-T1	832.09	1.827	936.91	1.693	3.44	0.622	20.89	1.271
C-Rb-T1	820.41	1.801	922.61	1.667	4.05	0.732	24.16	1.470
C-H-T2	525.75	1.154	731.98	1.322	4.99	0.902	23.93	1.445
C-N-T2	859.07	1.886	1018.3	1.840	3.81	0.689	17.90	1.089

**Table 7:** Comparison of bearing capacities of specimens before and after fire.

No.	Test Results			Calculation Results-EC4 [44]		Calculation Results-CECS [45]	
	$N_{u,e}$/kN	$N_{u,e}^{f}$/kN	$N_{u,e}^{f}$/$N_{u,e}$	$N_{u,c}$/kN	$N_{u,e}^{f}$/$N_{cr,c}$	$N_{u,c}$/kN	$N_{u,e}^{f}$/$N_{cr,c}$
C-N-T1	936.9	865.4	0.923	903.2	0.958	867.5	0.998
C-Ra-T1	935.6	777.3	0.831	903.2	0.861	867.5	0.896
C-Rb-T1	922.3	761.6	0.826	903.2	0.843	867.5	0.878
C-N-T2	1020.1	966.1	0.947	989.6	0.976	943.8	1.024
C-Ra-T2	-	924.2	-	989.6	0.934	943.8	0.979
C-Rb-T2	-	965.6	-	989.6	0.967	943.8	1.023

## Abbreviations

The following abbreviations are used in this manuscript:

CFST	Concrete filled steel tube
RAC	Recycled aggregate concrete
RCA	Recycled concrete aggregate
FAC	Fine aggregate concrete
RACFST	Recycled aggregate concrete-filled steel tube
RP	Replacement percentage
*MV*	Measured value
*RV*	Relative value

## References

[1] Xiao J.Z., Li J.B., Lan Y. (2003). Research on recycled aggregate concrete—A review. Concrete.

[2] Liu W., Cao W., Zhang J. (2016). Seismic Performance of Composite Shear Walls Constructed Using Recycled Aggregate Concrete and Different Expandable Polystyrene Configurations. Materials.

[3] Safiuddin M., Alengaram U.J., Rahman M.M. (2013). Use of recycled concrete aggregate in concrete: A review. J. Civ. Eng. Manag..

[4] Topçu I.B. (1997). Physical and mechanical properties of concretes produced with waste concrete. Cem. Concr. Res..

[5] Sagoe-Crentsil K.K., Brown T., Taylor A.H. (2001). Performance of concrete made with commercially produced coarse recycled concrete aggregate. Cem. Concr. Res..

[6] Xiao J.Z., Li J.B., Zhang C. (2005). Mechanical properties of recycled aggregate concrete under uniaxial loading. Cem. Concr. Res..

[7] Evangelista L., de Brito J. (2007). Mechanical behaviour of concrete made with fine recycled concrete aggregates. Cem. Concr. Compos..

[8] Rahal K. (2007). Mechanical properties of concrete with recycled coarse aggregate. Build. Environ..

[9] Evangelista L., de Brito J. (2010). Durability performance of concrete made with fine recycled concrete aggregates. Cem. Concr. Compos..

[10] Behnood A., Olek J., Glinicki M.A. (2015). Predicting modulus elasticity of recycled aggregate concrete using M5′ model tree algorithm. Constr. Build. Mater..

[11] Gales J., Parker T., Cree D., Green M. (2016). Fire Performance of Sustainable Recycled Concrete Aggregates: Mechanical Properties at Elevated Temperatures and Current Research Needs. Fire Technol..

[12] Li L., Xiao J.Z., Poon C.S. (2016). Dynamic compressive behavior of recycled aggregate concrete. Mater. Struct..

[13] Bendimerad A.Z., Roziere E., Loukili A. (2016). Plastic shrinkage and cracking risk of recycled aggregates concrete. Constr. Build. Mater..

[14] Dong H., Cao W., Bian J., Zhang J. (2014). The Fire Resistance Performance of Recycled Aggregate Concrete Columns with Different Concrete Compressive Strengths. Materials.

[15] Sato R., Maruyama I., Sogabe T., Sog M. (2007). Flexural behavior of reinforced recycled concrete beams. J. Adv. Concr. Technol..

[16] Kang T.H.-K., Kim W., Kwak Y.-K., Hong S.-G. (2014). Flexural Testing of Reinforced Concrete Beams with Recycled Concrete Aggregates. ACI Struct. J..

[17] Cao W.L., Zhang J., Dong H.Y. (2014). Experimental research on flexural performance of high strength recycled aggregate concrete slabs with steel bar truss. J. Build. Struct..

[18] Cao W.L., Liu Q., Zhang J.W. (2011). Seismic Experiment and analysis of low-rise recycled concrete shear wall. J. Beijing Univ. Technol..

[19] Cao W.L., Xu T.G., Liu Q. (2009). Experimental study on seismic performance of high-rise recycled aggregate concrete shear wall. World Earthq. Eng..

[20] Cao W.L., Cheng J., Zhang Y.B. (2015). Experiment of seismic behavior of low-rise recycled aggregate concrete shear wall with insulation modules and single layer of reinforcement. J. Build. Struct..

[21] Zhang J.W., Cao W.L., Meng S.B. (2014). Shaking table experimental study of recycled concrete frame-shear wall structures. Earthq. Eng. Eng. Vib..

[22] Zhang J.W., Dong H.Y., Cao W.L. (2016). Shaking Table Tests of Low-Rise Shear Walls Made of Recycled Aggregate Concrete. Struct. Eng. Int..

[23] Konno K., Sato Y., Kakuta Y. (1997). Property of recycled concrete column encased by steel tube subjected to axial compression. Trans. Jpn. Concr. Inst..

[24] Konno K., Sato Y., Kakuta Y. (1998). Mechanical property of recycled concrete under lateral confinement. Trans. Jpn. Concr. Inst..

[25] Johansson M., Gylltoft K. (2002). Mechanical Behavior of Circular Steel-Concrete Composite Stub Columns. J. Struct. Eng..

[26] Hu Y.M., Yu T., Teng J.G. (2011). FRP-Confined Circular Concrete-Filled Thin Steel Tubes under Axial Compression. J. Compos. Constr..

[27] Karantzikis M., Papanicolaou C.G., Antonopoulos C.P. (2005). Experimental investigation of nonconventional confinement for concrete using FRP. J. Compos. Constr..

[28] Rousakis T. (2013). Hybrid Confinement of Concrete by FRP Sheets and Fiber Ropes Under Cyclic Axial Compressive Loading. J. Compos. Constr..

[29] Triantafillou T.C., Papanicolaou C.G., Zissimopoulos P. (2006). Concrete confinement with textile-reinforced mortar jackets. ACI Struct. J..

[30] Choi E., Jeon J.S., Cho B.S. (2013). External jacket of FRP wire for confining concrete and its advantages. Eng. Struct..

[31] Rousakis T. (2014). Elastic Fiber Ropes of Ultrahigh-Extension Capacity in Strengthening of Concrete through Confinement. J. Mater. Civ. Eng..

[32] Anggawidjaja D., Ueda T., Dai J. (2006). Deformation capacity of RC piers by new fiber-reinforced polymer with large fracture strain. Cem. Concr. Compos..

[33] Rousakis T.C., Kouravelou K.B., Karachalios T.K. (2014). Effects of Carbon Nanotube Enrichment of Epoxy Resins on Hybrid FRP-FR Confinement of Concrete. Compos. B Eng..

[34] Dai J.G., Bai Y.L., Teng J.G. (2011). Behavior and Modeling of Concrete Confined with FRP Composites of Large Deformability. J. Compos. Constr..

[35] Yang Y.F., Han L.H. (2006). Experimental behaviour of recycled aggregate concrete filled steel tubular columns. J. Constr. Steel Res..

[36] Mohanraj E.K., Kandasamy S., Malathy R. (2011). Behaviour of steel tubular stub and slender columns filled with concrete using recycled aggregates. J. S. Afr. Inst. Civ. Eng..

[37] Chen Z.P., Zhang X.G., Xue J.Y. (2016). Analysis on aseismic performance and influence factors of recycled concrete filled circular steel-tube columns. Eng. Mech..

[38] Yang Y.F., Hou R. (2012). Experimental study and theoretical analysis on mechanical behavior recycled aggregate concrete filled steel tubular short column after high temperature. J. Disaster Prev. Mitig. Eng..

[39] Chen Z.P., Jing C.G., Xue J.Y. (2014). Study of mechanical behavior recycled aggregate concrete filled circular steel tube columns under eccentric loading after high temperature. Ind. Constr..

[40] Luo C.N., Zha X.X. (2015). Research on fire resistance of recycled concrete filled steel tubular columns. J. Build. Struct..

[41] China Standards Publication (2011). Recycled Coarse Aggregate for Concrete.

[42] China Standards Publication (2011). Code for Construction of Concrete Structures.

[43] China Standards Publication (2003). Metallic Materials-Tensile Testing at Ambient Temperature.

[44] Eurocodes (2004). Design of Composite Steel and Concrete Structures, Part 1.1: General Rules and Rules for Buildings.

[45] Trade Standards of China (1992). Design Regulation for the Fire Protection of Steel Structures.

